# Predictive model of delayed hyponatremia after endoscopic endonasal transsphenoidal resection of pituitary adenoma

**DOI:** 10.3389/fnhum.2025.1674519

**Published:** 2025-12-05

**Authors:** Hangyi Tan, Xianlong Miao, Yukang Pei, Faan Miao

**Affiliations:** Department of Neurosurgery, Department of Skull Base Oncology, Affiliated Hospital of Xuzhou Medical University, Xuzhou, Jiangsu, China

**Keywords:** pituitary adenoma, transsphenoidal surgery, delayed hyponatremia, nomograms, risk stratification

## Abstract

**Objective:**

This study aims to establish the risk factors and predictive model for the occurrence of delayed hyponatremia after endoscopic endonasal transsphenoidal resection of pituitary adenoma.

**Methods:**

Data from 155 patients who underwent endoscopic endonasal transsphenoidal resection of pituitary adenoma at the affiliated hospital of Xuzhou Medical University from January 2018 to May 2023 were analyzed. These patients were randomly divided into a training group (108 cases, 70%) and a validation group (47 cases, 30%). Univariate and Multivariate Logistic regression analysis were conducted on the training group to identify risk factors for delayed hyponatremia after surgery. A predictive model was established using R software and validated.

**Results:**

After conducting Univariate and Multivariate Logistic regression analysis, factors influencing the occurrence of delayed hyponatremia after endoscopic endonasal transsphenoidal resection of pituitary adenoma were identified as follows: elevated preoperative prolactin levels, higher preoperative suprasellar cistern height, and hyponatremia in the first 1–2 days after surgery. The area under the ROC curve for forecasting delayed postoperative hyponatremia (DPH) in training and validation sets was 0.943 and 0.959, respectively. The DCA curve indicated a higher benefit in clinical application.

**Conclusion:**

The risk prediction model for delayed hyponatremia after endoscopic endonasal transsphenoidal resection of pituitary adenoma, developed in this study, demonstrates favorable predictive performance. The nomogram can be utilized for early identification of high-risk individuals for DPH.

## 1 Introduction

Pituitary adenoma (PA) is a tumor that grows in the sellar region of the anterior pituitary gland. Its incidence ranks only after gliomas and meningiomas (Roncaroli and Giannini, 2025; Villa et al., 2025). Most pituitary adenomas can be removed through transsphenoidal surgery (TSS) using an endoscopic endonasal approach. This surgical technique not only avoids traction on brain tissue and cranial nerves but also maximizes tumor removal while reducing postoperative complications, thereby shortening hospital stays (van Furth et al., 2020).

Delayed postoperative hyponatremia (DPH) refers to hyponatremia occurring on or after the 3rd day following surgery (Yu et al., 2022). The incidence of DPH after transsphenoidal surgery for pituitary adenoma varies between 7.4% and 14.7% (Lee et al., 2021). Patients with hyponatremia may present with various clinical symptoms, and severe cases can lead to altered mental status, seizures, coma, and even death (Hong et al., 2021). Additionally, research suggests that DPH is a major risk factor for readmission within 30 days postoperatively for pituitary adenoma (Yu et al., 2022). The objective of this study is to explore the risk factors for DPH after endoscopic endonasal transsphenoidal resection of pituitary adenoma and to construct a predictive model to identify and screen high-risk patients, thereby assisting clinical decision-making.

## 2 Materials and methods

### 2.1 Patient cohort dataset details

**Figure 1:**
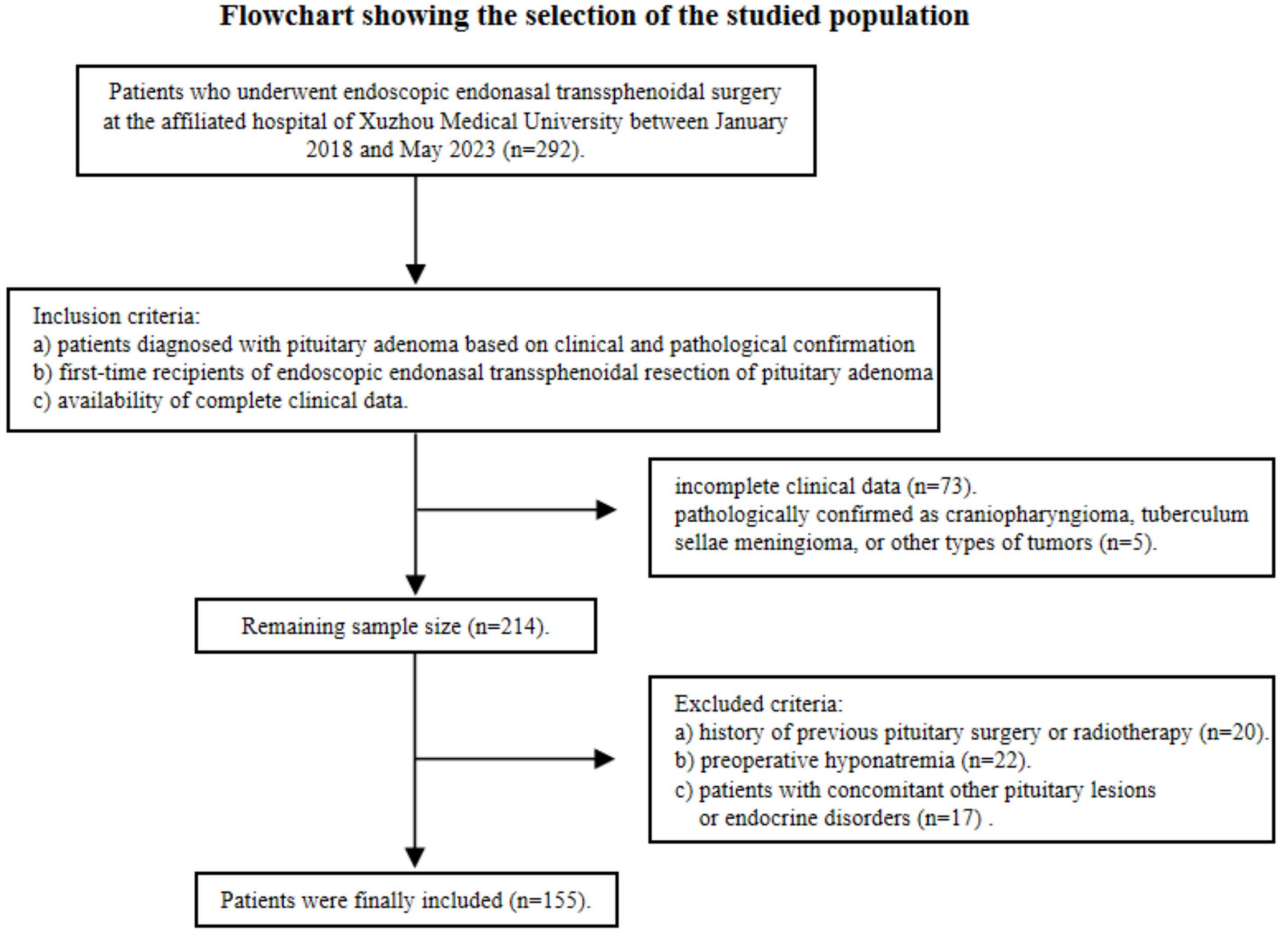
Flowchart showing the selection of the studied population.

A retrospective analysis was conducted on clinical data collected from 155 patients who underwent endoscopic endonasal transsphenoidal surgery at the affiliated hospital of Xuzhou Medical University between January 2018 and May 2023 (Figure 1). Inclusion criteria were: a) patients diagnosed with pituitary adenoma based on clinical and pathological confirmation; b) first-time recipients of endoscopic endonasal transsphenoidal resection of pituitary adenoma; c) availability of complete clinical data. Exclusion criteria were: a) history of previous pituitary surgery or radiotherapy; b) preoperative hyponatremia; c) patients with concomitant other pituitary lesions or endocrine disorders. All surgeries were performed by the same surgical team. The study was approved by the Ethics Committee of Xuzhou Medical University (Approval No. XYFY2023-KL250-01), and all patients gave informed consent.

### 2.2 Research data

Collected and compiled clinical data, including demographic information, surgical procedures, and postoperative outcomes. Laboratory data encompassed hormone levels before and after surgery: adrenocorticotropic hormone (ACTH), cortisol, prolactin (PRL), growth hormone (GH), thyroid-stimulating hormone, and insulin-like growth factor, as well as serum sodium levels (preoperatively and postoperatively for the first 1–3 days; in case of hyponatremia, daily monitoring until normalization, otherwise every 3 days). Imaging data included pre- and postoperative pituitary MRI scans (plain and enhanced scans) for evaluating tumor size, Knosp grading, pre- and postoperative angles of pituitary stalk deviation, height increase of the diaphragma sellae before and after surgery, and extent of tumor resection. To ensure objectivity and reproducibility, the preoperative and postoperative MRI images were independently evaluated by two senior neuroradiologists (with 10 and 8 years of experience, respectively) who were blinded to the patients’ clinical outcomes (i.e., whether they developed DPH). Any discrepancies in their measurements were resolved through discussion until a consensus was reached.

### 2.3 Diagnostic criteria and definitions

**Figure 2:**
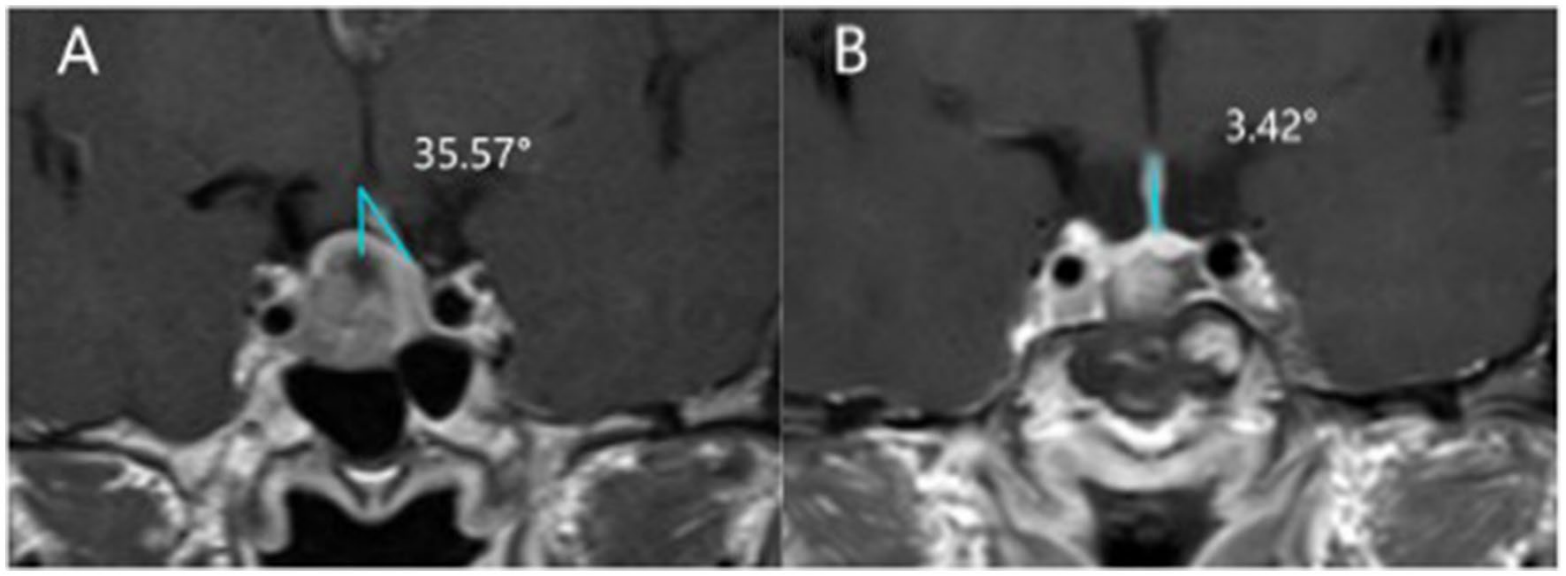
The difference in pituitary stalk deviation angle before and after transsphenoidal surgery. **(A)** Before surgery, the pituitary stalk deviates 35.57° to the right. **(B)** After surgery, the tumor was totally removed, and the pituitary stalk deviation angle was 3.42°.

**Figure 3:**
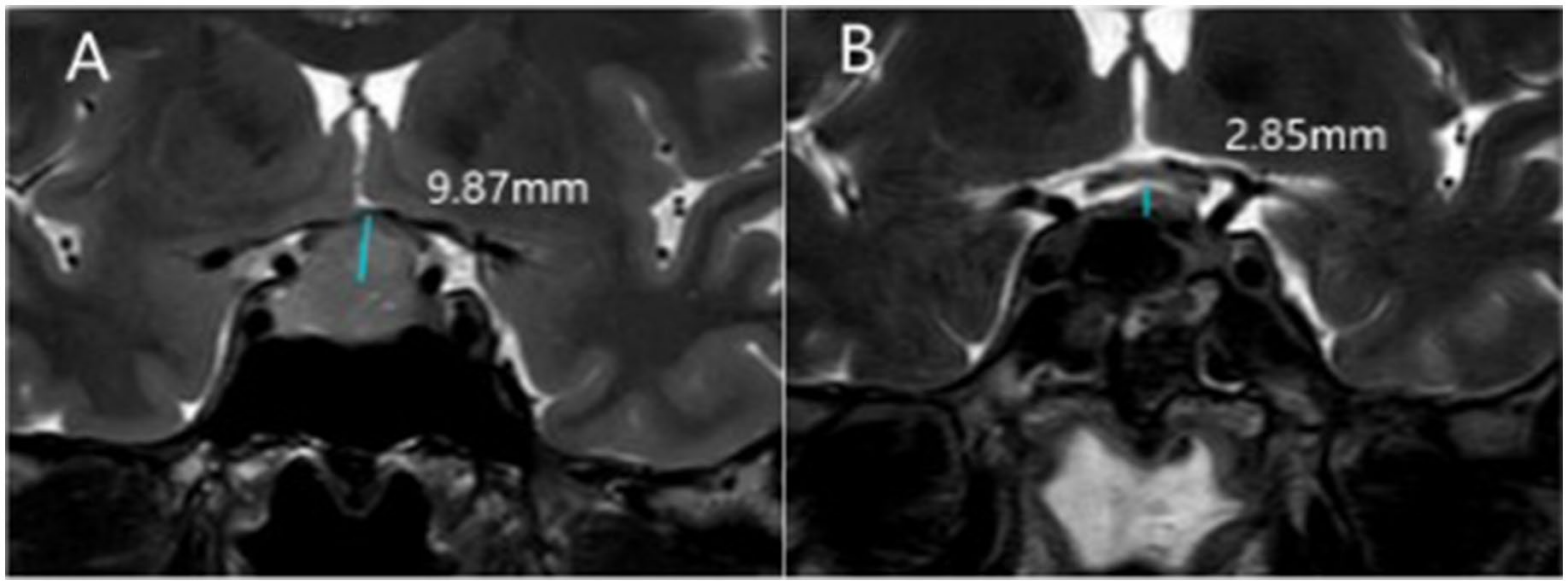
Changes in the diaphragma sellae (DS) before and after transsphenoidal surgery. **(A)** Preoperative elevation of DS was 9.87 mm. **(B)** After resection of the tumor, the elevation of DS was 2.85 mm.

Considering potential variations in reference values across different laboratories (Miller et al., 2023), low serum sodium concentration is defined as below 137 mmol/L based on the laboratory settings of our institution. Upon admission, all patients undergo radiological examination to observe tumor location and its relationship with surrounding tissues, classified into Knosp grades 0–4 (Knosp et al., 1993). The postoperative MRI scans were routinely performed on postoperative day 3. The angle of deviation of the pituitary stalk is recorded on T1 + C scans, defined as the angle at which the pituitary stalk deviates from the midline at its point of origin (Lin et al., 2022) (Figure 2). On T2W1 scans, The height of the diaphragma sellae was measured on coronal T2-weighted images because we found this plane best visualizes the contour of the diaphragma sellae relative to the sella turcica.the height of the diaphragma sellae elevation (the distance between the plane where the elevation of the diaphragma sellae begins and the plane of the highest point of the sellae) is calculated (Figure 3). Tumor volume is calculated using the simplified ellipsoid volume formula *V* = ABC/2.

Identifying the precise starting point of the elevation could be challenging in some cases, particularly when there was significant postoperative change or artifact. To ensure consistency, the two neuroradiologists used predefined anatomical landmarks (the tuberculum sellae and dorsum sellae) and referenced the preoperative images for comparison. The inter-rater reliability for this measurement, calculated using the intraclass correlation coefficient (ICC), was (0.68), indicating excellent agreement.

In our study, postoperative diabetes insipidus was diagnosed based on the following criteria: persistent polyuria, defined as a urine output exceeding 300 mL per hour for two consecutive hours or a total daily urine volume greater than 3 L; concurrent laboratory evidence of hyperosmolality (serum osmolality >300 mOsm/kg) or hypernatremia (serum sodium >145 mmol/L); and the clinical requirement for therapeutic intervention with desmopressin (DDAVP).

### 2.4 Statistical analysis

The data analysis was performed using SPSS 25.0 software. Patients were randomly divided into two groups: a training group (108 cases, 70%) and a validation group (47 cases, 30%). Continuous variables were expressed as mean ± standard deviation ( $\bar{x}\;$ ± s), and differences between the two groups were compared using the *t*-test. Categorical variables were presented as case numbers, and intergroup comparisons were made using the chi-square test (*χ*^2^ test). Non-parametric tests were used for comparing ordinal data. Logistic regression analysis was conducted to determine independent risk factors in the training group. Differences were considered statistically significant when *p*-values were less than 0.05.

The identified independent risk factors were then imported into R software (version 4.3.3) for analysis. Based on this, a nomogram predictive model was constructed for the training group. The predictive performance of this model was evaluated in the validation group by calculating the area under the receiver operating characteristic (ROC) curve (AUC), calibration curve, and decision curve analysis (DCA). These assessments were used to evaluate the predictive ability of the nomogram generated from the training group.

To investigate the impact of tumor functional status on the predictive model, we conducted two supplementary analyses. First, a subgroup analysis was performed exclusively in patients with non-functional pituitary adenomas (NFPA) to validate the predictive factors in the context of the “pituitary stalk effect.” Second, in the full cohort comprising all patients, we incorporated “tumor functional status (functioning vs. non-functioning)” as a covariate in a multivariable logistic regression model as part of a sensitivity analysis, to assess the robustness of the core predictors after adjusting for tumor type.

## 3 Results

### 3.1 The basic characteristics of the cases

The study retrospectively analyzed 155 patients who underwent endoscopic endonasal transsphenoidal resection of pituitary adenoma at the affiliated hospital of Xuzhou Medical University from January 2018 to May 2023. Among them, 50 patients (32%) developed delayed hyponatremia. These patients were randomly divided into a training group (108 cases) and a validation group (47 cases) at a ratio of 7:3. In the training group, 39 patients developed delayed hyponatremia, while in the validation group, there were 11 cases. A comparison of clinical data between the training and validation groups showed no significant differences (Table 1).

**Table 1:** Comparison of the characteristics between the training and validation cohorts.

Factors	Training group (*n* = 108)	Validation group (*n* = 47)	*t*/*χ*^2^/*Z*	p*
Age, years	47.9 ± 12.5	50.9 ± 15.6	1.617	0.106
Sex				
Male	51 (47.2)	27 (57.4)	1.166	0.243
Female	57 (52.8)	20 (42.6)		
Dizziness and headache	64 (59.3)	26 (55.3)	0.455	0.649
Visual damage and optic filed defect	51 (47.2)	23 (48.9)	0.196	0.845
Altered menstrual period	9 (8.3)	6 (12.8)	0.855	0.392
Galactorrhea	1 (0.9)	1 (2.1)	0.607	0.544
Changes in sexual function	0 (0.0)	1 (2.1)	1.516	0.130
Acromegalia	4 (3.7)	3 (6.4)	0.736	0.462
Preoperative ACTH (pg/mL)	25.6 (19.8, 34.8)	29.1 (19.2,35.6)	1.094	0.274
Preoperative cortisol (ug/dL)	9.9 (7.5, 13.1)	11.2 (7.2, 15.4)	0.938	0.348
Preoperative PRL (ng/mL)	24.6 (15.7, 256.8)	22.9 (12.5, 40.1)	1.308	0.108
Preoperative GH (ng/mL)	0.6 (0.2, 1.6)	0.3 (0.1, 0.9)	1.829	0.067
Postoperative ACTH (pg/mL)	22.4 (15.6, 35.9)	28.3 (16.3, 35.9)	1.633	0.102
Postoperative cortisol (ug/dL)	14.8 (7.9, 18.7)	18.6 (8.9, 25.1)	2.423	0.015
Postoperative PRL (ng/mL)	21.3 (9.5, 139.5)	19.1 (7.0, 66.4)	1.030	0.303
Postoperative GH (ng/mL)	0.9 (0.6, 1.6)	0.8 (0.5, 1.6)	0.152	0.879
Preoperative sodium levels (mmol/L)	141.5 (139.0, 142.3)	141.5 (140.5, 142.5)	1.712	0.087
Sodium levels 1–2 days after surgery (mmol/L)	139.7 (136.5, 141.9)	141.6 (139.3, 142.8)	1.937	0.053
Sodium levels 3 days after surgery (mmol/L)	139.5 (134.7, 141.9)	141.1 (139.4, 143.0)	2.934	0.003
Maximum tumor diameter (mm)	25 (16, 34)	20 (16, 25)	2.548	0.011
Tumor volume (cm^3^)	4.7 (2.2, 10.0)	3.2 (2.0, 5.4)	2.067	0.039
Preoperative pituitary stalk deviation angle (°)	37.0 (20.0, 45.7)	29.2 (20.1, 38.9)	1.681	0.093
Postoperative pituitary stalk deviation angle (°)	20.0 (2.1, 29.1)	12.3 (0.0, 22.0)	1.681	0.120
Preoperative elevation of the diaphragma sellae (mm)	13.5 (5.0, 20.0)	8.0 (5.0, 15.0)	1.719	0.097
Postoperative elevation of the diaphragma sellae (mm)	4.5 (0.0, 10.0)	3.0 (0.0, 8.0)	1.824	0.068
Knosp grade				
Grade 0–2	62 (57.5)	36 (76.6)	1.659	0.097
Grade 3–4	46 (42.5)	11 (23.4)		
Extent of tumor resection				
Total resection	92 (85.2)	44 (93.6)	1.486	0.137
Subtotal resection	14 (13.0)	3 (6.4)		
Partial resection	2 (1.9)	0 (0.0)		
Intraoperative CSF leakage	14 (13.0)	7 (14.9)	0.322	0.748
Postoperative diabetes insipidus	49 (45.4)	12 (25.5)	2.316	0.021
Hyponatremia 1–2 days after surgery	32 (29.6)	7 (14.9)	1.937	0.053
Hyponatremia 3 days after surgery	39 (36.1)	11 (23.4)	2.934	0.121

ACTH, adrenocorticotropic hormone; PRL, prolactin; GH, growth hormone; CSF, cerebral spinal fluid. *p-values between training and validation group.

### 3.2 Univariate and multivariate logistic regression analysis of delayed hyponatremia

In the training cohort, univariate analysis was performed to compare clinical variables between the delayed hyponatremia group (*n* = 39) and the normal sodium group (*n* = 69). The results revealed statistically significant differences (*p* < 0.05) in the following factors: preoperative and postoperative PRL levels, maximum tumor diameter, tumor volume, preoperative and postoperative pituitary stalk deviation angle, preoperative and postoperative elevation of the diaphragma sellae, Knosp grade, preoperative serum sodium levels, presence of hyponatremia on postoperative days 1–2, and occurrence of postoperative diabetes insipidus. These findings suggest that these variables are associated with the development of delayed hyponatremia.

Subsequently, variables with *p* < 0.05 in the univariate analysis were included in a multivariate logistic regression model to identify independent risk factors. The analysis identified three independent predictors for delayed hyponatremia following transsphenoidal surgery for pituitary adenoma: elevated preoperative PRL levels (OR = 1.010, 95% CI: 1.003–1.018, *p* = 0.005), greater preoperative elevation of the diaphragma sellae (OR = 1.651, 95% CI: 1.190–2.290, *p* = 0.003), and the presence of hyponatremia on postoperative days 1–2 (OR = 32.65, 95% CI: 2.188–487.28, *p* = 0.011). These factors remained significantly associated with the outcome after adjusting for other covariates. Detailed results of the univariate and multivariate analyses are presented in Table 2.

**Table 2:** Univariate and multivariate logistic regression analysis of delayed hyponatremia after transsphenoidal surgery for pituitary adenomas [*n* (%)].

Univariate analysis					Logistic regression analysis		
Factors	Delayed hyponatremia group (*n* = 39)	Normal sodium group (*n* = 69)	*t*/*χ*^2^/*Z*	p*	Odds ratio	95%CI	p*
Preoperative PRL (ng/mL)	506.7 (24.3, 1897.5)	21.1 (13.9, 35.9)	5.280	0.000	1.010	1.003–1.018	0.005
Postoperative PRL (ng/mL)	111.9 (10.6, 323.3)	18.6 (9.2, 27.6)	3.682	0.000			
Maximum tumor diameter (mm)	34 (26, 41)	20 (14, 27)	5.536	0.000			
Tumor volume (cm^3^)	10.0 (5.4, 16.5)	3.8 (1.3, 6.2)	5.149	0.000			
Preoperative pituitary stalk deviation angle (°)	45.6 (40.1, 56.4)	28.7 (11.4, 39.7)	5.663	0.000			
Postoperative pituitary stalk deviation angle (°)	24.1 (17.6, 32.8)	12.7 (0.0, 24.6)	3.691	0.000			
Preoperative elevation of the diaphragma sellae (mm)	20 (15, 25)	8 (5, 15)	6.062	0.000	1.651	1.190–2.290	0.003
Postoperative elevation of the diaphragma sellae (mm)	7 (3, 17)	3.0 (0.0, 8.0)	3.697	0.000			
Knosp grade							
Grade 0–2	12 (30.7)	50 (72.5)	4.799	0.000			
Grade 3–4	27 (69.3)	19 (27.5)					
Postoperative diabetes insipidus	30 (45.4)	19 (25.5)	4.929	0.000			
Preoperative sodium levels (mmol/L)	140.2 (138.0, 141.7)	141.3 (139.7, 142.3)	2.329	0.020			
Hyponatremia 1–2 days after surgery	24 (81)	8 (8.3)	5.434	0.000	32.65	2.188–487.28	0.011
Tumor functional status	18 (46.1)	20 (28.9)	0.956	0.328	0.807	0.314–2.074	0.656

To further validate the predictive factors, particularly in the context of the “pituitary stalk effect,” a subgroup analysis was conducted exclusively on patients with non-functional pituitary adenomas (NFPA). The results confirmed that elevated preoperative PRL (OR = 1.002, 95% CI: 1.001–1.004, *p* = 0.004), greater preoperative elevation of the diaphragma sellae (OR = 1.202, 95% CI: 1.063–1.354, *p* = 0.003), and early postoperative hyponatremia (OR = 6.647, 95% CI: 1.693–26.098, *p* = 0.007) remained significant predictors in this subgroup (Table 3).

**Table 3:** Univariate and multivariate logistic regression analysis of delayed hyponatremia after transsphenoidal surgery for NFPA [*n* (%)].

Univariate analysis					Logistic regression analysis		
Factors	Delayed hyponatremia group (*n* = 21)	Normal sodium group (*n* = 49)	*t*/*χ*^2^/*Z*	p*	Odds ratio	95%CI	p*
Preoperative PRL (ng/mL)	25.6 (16.8, 156.4)	18.9 (13.2, 28.5)	2.134	0.033	1.002	1.001–1.004	0.004
Postoperative PRL (ng/mL)	21.8 (10.5, 89.3)	16.3 (8.9, 24.7)	3.682	0.061			
Maximum tumor diameter (mm)	26 (20, 35)	18 (14, 25)	3.245	0.000			
Tumor volume (cm^3^)	7.8 (4.2, 12.6)	3.2 (1.5, 5.8)	3.567	0.000			
Preoperative pituitary stalk deviation angle (°)	38.7 (28.5, 49.2)	25.4 (10.8, 36.9)	5.663	0.000			
Postoperative pituitary stalk deviation angle (°)	18.9 (12.4, 27.6)	10.3 (0.0, 21.5)	2.674	0.007			
Preoperative elevation of the diaphragma sellae (mm)	16 (11, 22)	7 (4, 13)	4.235	0.000	1.202	1.063–1.354	0.003
Postoperative elevation of the diaphragma sellae (mm)	5.8 (2.3, 12.1)	2.5 (0.0, 7.0)	3.697	0.000			
Postoperative diabetes insipidus	5 (23.8)	6 (12.2)	1.56	0.212			
Preoperative sodium levels (mmol/L)	140.8 (138.5, 142.1)	141.5 (139.9, 142.6)	1.654	0.098			
Hyponatremia 1–2 days after surgery	12 (57.1)	10 (20.4)	9.87	0.002	6.647	1.693–26.098	0.007

### 3.3 Development of prediction model in the training cohort

**Figure 4:**
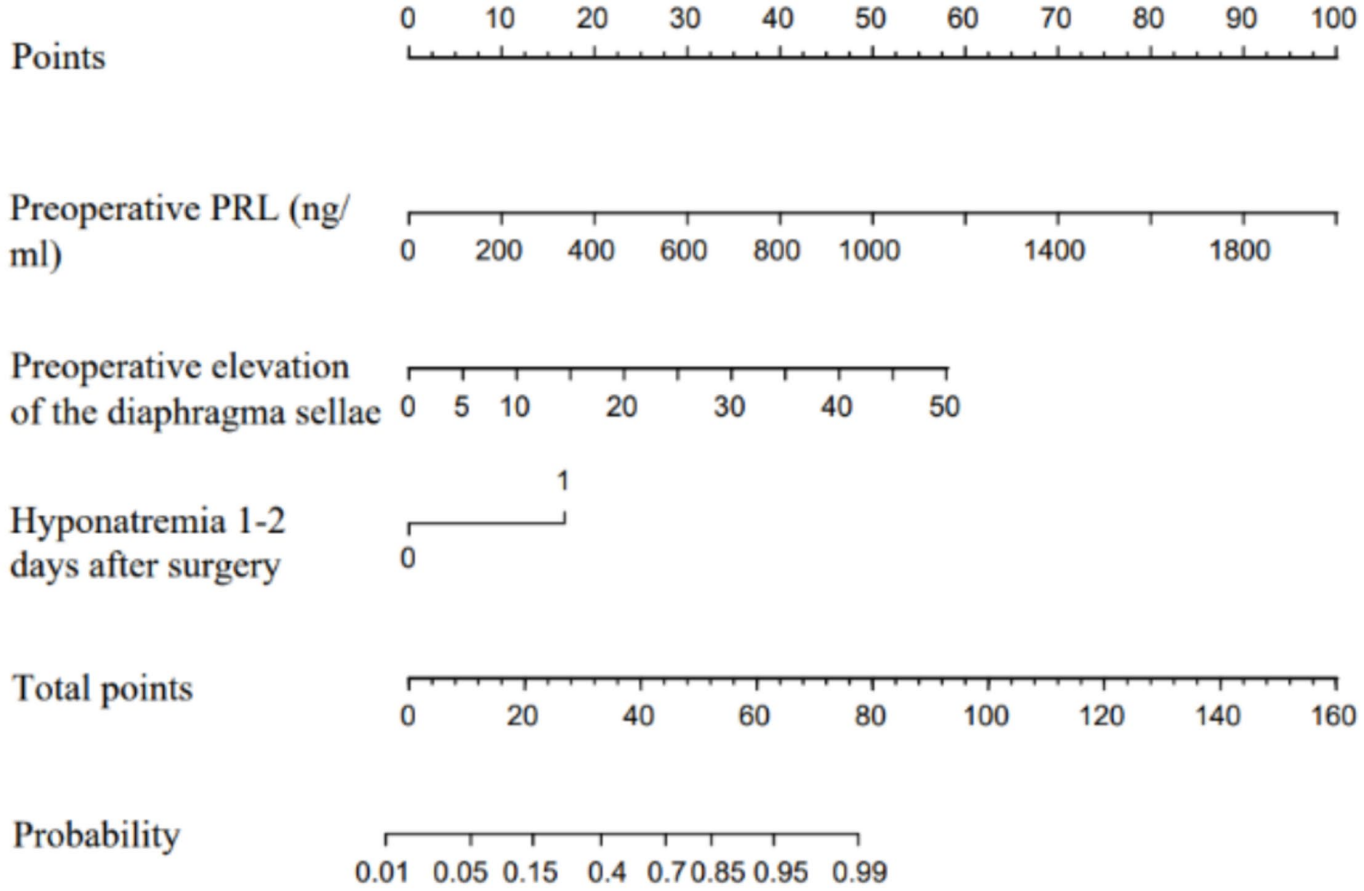
Nomogram of delayed hyponatremia after transsphenoidal adenoma surgery. The predictor points can be found on the uppermost point scale that correspond to each patient variable and can be added up. The total points projected to the bottom scale indicate the risk of delayed hyponatremia (for hyponatremia 1–2 days after surgery, 0 means “No,” 1 means “Yes”).

Using R software (version 4.3.3), a predictive model was constructed based on the three variables selected from the logistic regression analysis conducted on the training set. This model is represented by a nomogram, which is used to estimate the likelihood of delayed hyponatremia occurrence after endoscopic transsphenoidal surgery for pituitary adenoma. The nomogram assigns a composite score based on the parameter values in the nomogram, which is then mapped to corresponding risk levels to estimate the risk of developing hyponatremia postoperatively (Figure 4).

### 3.4 Validation of the nomogram for delayed hyponatremia

**Figure 5:**
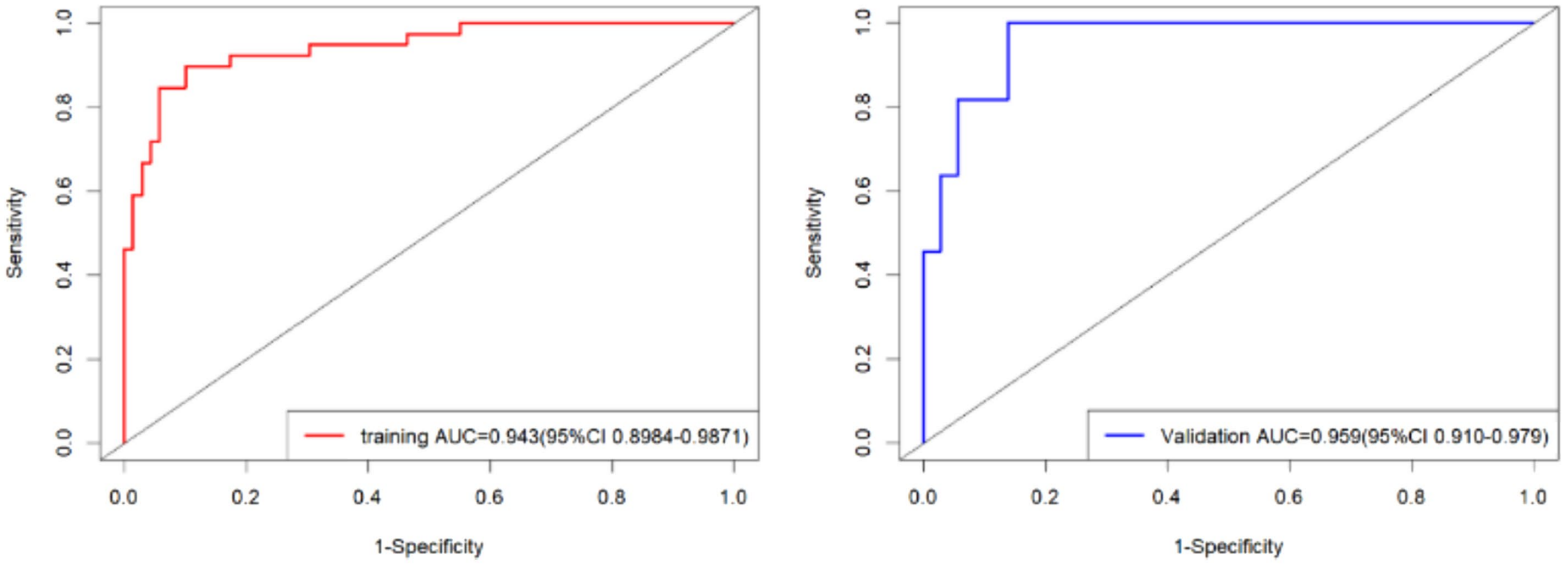
The ROC curves of the nomogram model (left: training set, right: validation set).

**Figure 6:**
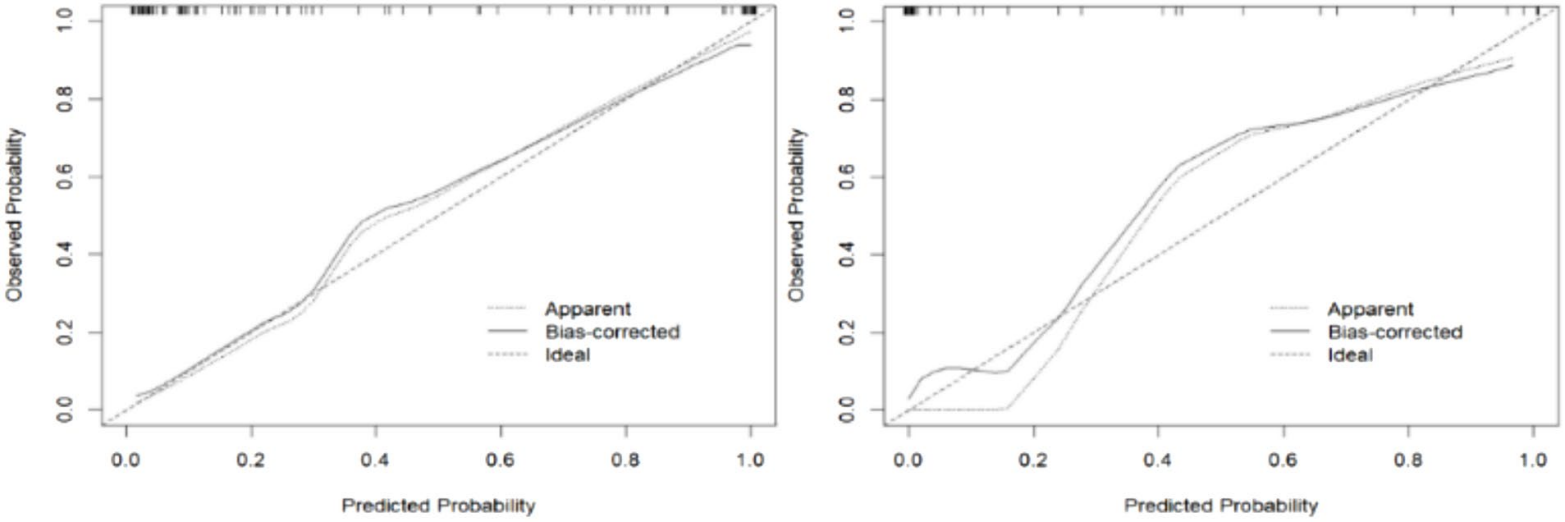
The calibration curves of the nomogram model (left: training set, right: validation set).

**Figure 7:**
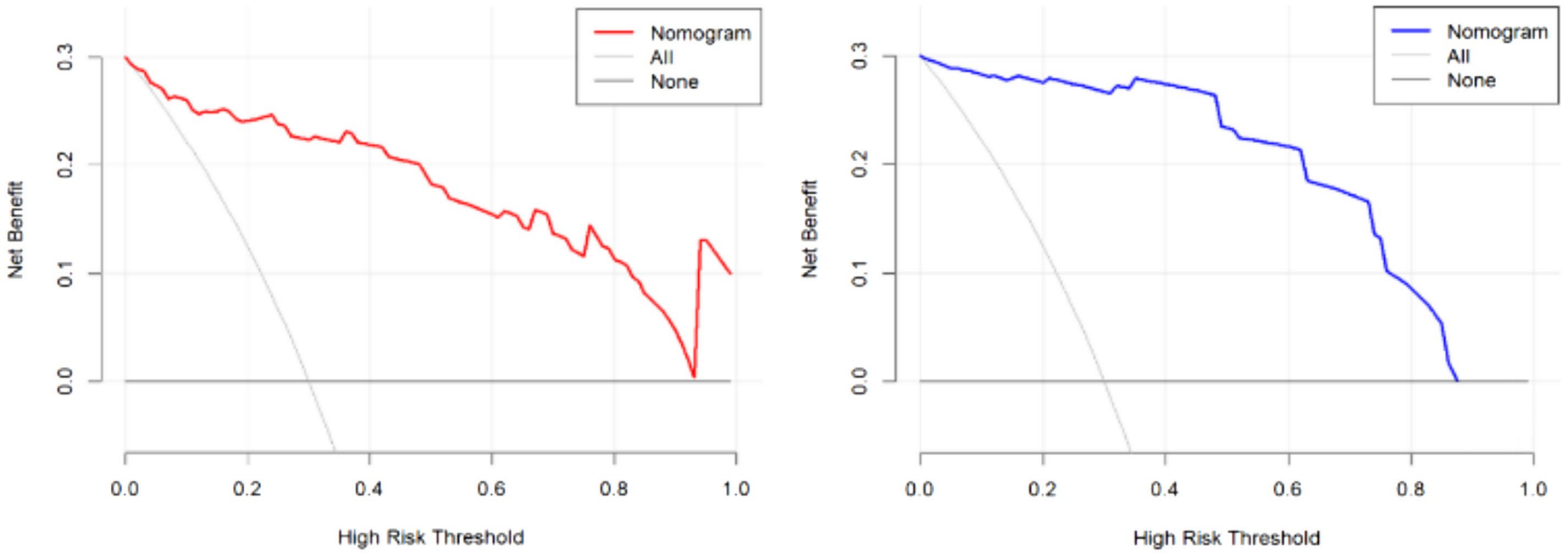
The DCA (decision curve analysis) curves (left: training set, right: validation set).

External validation of the model was performed using data from the validation group comprising 47 patients. The area under the ROC curve for the nomogram model in the training set was 0.943 (95% CI: 0.898–0.987), while in the validation set, it was 0.959 (95% CI: 0.910–0.979) (Figure 5). These results indicate that the model has good discriminative ability on both internal and external data. In the calibration curve analysis, the predicted results of the model closely matched the observed outcomes in both groups, demonstrating a high level of fit (Figure 6). Additionally, DCA revealed that the application of this model within the threshold range of 0.01–0.93 (training group) and 0.01–0.87 (validation group) could lead to improved clinical utility, indicating its practical value in the clinical setting (Figure 7). Additional validation performed specifically on the NFPA subgroup yielded an AUC of 0.867 (95% CI: 0.783–0.951). The model’s performance metrics were 78.3% for sensitivity, 82.9% for specificity, and 81.8% for accuracy, thereby affirming its retained predictive capability in a cohort exclusively composed of non-functioning adenomas.

### 3.5 Sensitivity analysis

To assess the robustness of the model, a sensitivity analysis incorporating tumor functional status as a covariate confirmed that the three original predictors remained statistically significant: preoperative prolactin (OR 1.002, 95% CI: 1.001–1.004, *p* = 0.004), preoperative sella turcica elevation (OR 1.200, 95% CI: 1.063–1.354, *p* = 0.003), and postoperative hyponatremia on the first 1–2 days (OR 6.647, 95% CI: 1.693–26.098, *p* = 0.007). Tumor functional status itself was not a significant predictor in the model (OR 0.807, 95%CI: 0.314–2.074, *p* = 0.656). These results confirm that the original predictors are robust and not significantly affected by tumor functional status.

## 4 Discussion

Delayed postoperative hyponatremia (DPH), a common cause of unplanned readmission after transsphenoidal surgery (TSS) for pituitary adenoma, is primarily attributed to the syndrome of inappropriate antidiuretic hormone secretion (SIADH), often triggered by surgical manipulation of the pituitary stalk (Klaassen et al., 2025; Bernardi et al., 2023; Snyder et al., 2022; Blair et al., 2017). This study identified three independent predictors for DPH: preoperative hyperprolactinemia, elevated diaphragma sellae height, and postoperative hyponatremia on the first 1–2 days.

The association of hyperprolactinemia with DPH aligns with prior findings in NFP (Huang et al., 2022). This supports the “pituitary stalk effect” as a key mechanism, where stalk compression reduces dopaminergic inhibition, elevating prolactin and indicating a predisposition to surgical injury and subsequent ADH dysregulation (Abeledo-Machado et al., 2023; Skinner, 2009). Similarly, a high preoperative diaphragma sellae suggests significant tumor mass effect. Its resection leads to a rapid reduction in intrasellar pressure, potentially causing mechanical traction on the stalk and precipitating SIADH (Kinoshita et al., 2022; Lin et al., 2022). Similarly, changes in the pituitary stalk deviation angle can also predict the occurrence of DPH after TSS (Lin et al., 2021; Lin et al., 2021; Rutland et al., 2020). Although pituitary stalk deviation angle changes were not an independent predictor in our cohort—possibly due to the confounding effect of early postoperative packing material on MRI assessment—the anatomic distortion captured by diaphragma sellae elevation proved highly informative. As a result, changes in the pituitary stalk may not be significant when pituitary MRI is performed approximately 3 days postoperatively.

In our study, patients who developed postoperative hyponatremia on the first 1–2 days had an approximately 32.6-fold increased risk of DPH. Yoon et al. (2019) found that patients with serum sodium concentration <138 mmol/L within 1–2 days after TSS had approximately 2.8 times higher risk of developing delayed hyponatremia, and similar results were reported by Krogh et al. (2018), consistent with other series. While common causes like adrenal insufficiency or DDAVP overdose were clinically excluded in our patients, undetermined pathophysiological factors may contribute to this marked association. Therefore, while close monitoring of early serum sodium is crucial, the interpretation of hyponatremia during this period warrants caution, and future studies are needed to elucidate its specific causes.

The predictive model demonstrated outstanding discriminative ability, with AUCs of 0.943 and 0.959 in the training and validation sets, respectively, significantly exceeding the conventional threshold for excellent performance (0.80–0.89) (Wu et al., 2020). This high predictive accuracy may be attributed to the synergistic combination of these predictors, which collectively capture key endocrine, anatomical, and early metabolic disruptions in DPH pathogenesis. Subgroup and sensitivity analyses confirmed the robustness of these predictors in both NFPA-only and mixed cohorts, underscoring the model’s generalizability across adenoma subtypes. While the single-center, standardized protocol may have enhanced internal validity, external validation is the necessary next step.

## 5 Limitations and future directions

This study has several limitations. Its single-center, retrospective design and limited sample size preclude external validation of our model. Furthermore, while our model incorporates key clinical predictors, it does not include emerging molecular classifications of pituitary neuroendocrine tumors (PitNETs) (Batchu et al., 2024) or patient-specific factors such as frailty, which is known to influence surgical outcomes (Findlay et al., 2025). Although internal validation via subgroup and sensitivity analyses confirmed the robustness of our findings, future prospective, multicenter studies are essential to validate the model externally and to refine it by incorporating molecular and frailty metrics.

## 6 Conclusion

In conclusion, we developed and validated a novel nomogram that accurately predicts the risk of DPH following endoscopic transsphenoidal surgery for pituitary adenoma. The model, incorporating preoperative prolactin, preoperative elevation of the diaphragma sellae, and early postoperative hyponatremia, demonstrated excellent discriminative ability upon internal and external validation. This tool shows significant potential for the early identification of high-risk patients, thereby facilitating timely interventions to mitigate the incidence of DPH and improve postoperative outcomes.

## Data Availability

The raw data supporting the conclusions of this article will be made available by the authors, without undue reservation.

## References

[ref1] Abeledo-Machado A. Bornancini D. Peña-Zanoni M. Camilletti M. A. Faraoni E. Y. Díaz-Torga G. (2023). Sex-specific regulation of prolactin secretion by pituitary activins in postnatal development. J. Endocrinol. 258:e230020. doi: 10.1530/JOE-23-0020, 37399522

[ref2] Batchu S. Diaz M. J. Patel A. Reddy A. Lucke-Wold B. (2024). Transcriptome-derived ligand-receptor interactome of major PitNET subgroups. J. Neurol. Surg. B Skull Base 85, 340–346. doi: 10.1055/a-2088-6594, 38966297 PMC11221903

[ref3] Bernardi S. Zorat F. Calabrò V. Faustini Fustini M. Fabris B. (2023). A case of cerebral salt wasting syndrome in a patient with central diabetes insipidus and status epilepticus. J. Endocrinol. Investig. 46, 1275–1277. doi: 10.1007/s40618-023-02053-z, 36932301

[ref4] Blair E. T. Clemmer J. S. Harkey H. L. Hester R. L. Pruett W. A. (2017). Physiologic mechanisms of water and electrolyte disturbances after transsphenoidal pituitary surgery. World Neurosurg. 107, 429–436. doi: 10.1016/j.wneu.2017.07.175, 28797976 PMC5800790

[ref5] Findlay M. C. Rennert R. C. Lucke-Wold B. Couldwell W. T. Evans J. J. Collopy S. . (2025). Impact of frailty on surgical outcomes of patients with Cushing disease using the multicenter registry of adenomas of the pituitary and related disorders registry. Neurosurgery 96, 386–395. doi: 10.1227/neu.0000000000003090, 39813068

[ref6] Hong Y. G. Kim S. H. Kim E. H. (2021). Delayed hyponatremia after transsphenoidal surgery for pituitary adenomas: a single institutional experience. Brain Tumor Res Treat 9, 16–20. doi: 10.14791/btrt.2021.9.e5, 33913267 PMC8082282

[ref7] Huang Y. Wang M. Wu J. Lin K. Wang S. Zhang F. (2022). Risk factors for delayed postoperative hyponatremia in patients with non-functioning pituitary adenomas undergoing transsphenoidal surgery: a single-institution study. Front. Neurol. 13:945640. doi: 10.3389/fneur.2022.945640, 35928122 PMC9343797

[ref8] Kinoshita Y. Taguchi A. Tominaga A. Arita K. Yamasaki F. (2022). Predictive factors for recovery from adult growth hormone deficiency after transsphenoidal surgery for nonfunctioning pituitary adenoma. J. Neurosurg. 137, 629–634. doi: 10.3171/2021.10.JNS211999, 35171826

[ref9] Klaassen D. Mok S. Hwang J. Y. Blount S. L. Williams K. J. Fong B. M. . (2025). Post-operative fluid restriction to prevent delayed hyponatremia after endoscopic transsphenoidal surgery. Neuro-Oncol.:noaf069. doi: 10.1093/neuonc/noaf069 PMC12417837 40084913

[ref10] Knosp E. Steiner E. Kitz K. Matula C. (1993). Pituitary adenomas with invasion of the cavernous sinus space: a magnetic resonance imaging classification compared with surgical findings. Neurosurgery 33, 610–617. doi: 10.1227/00006123-199310000-00008 8232800

[ref11] Krogh J. Kistorp C. N. Jafar-Mohammadi B. Pal A. Cudlip S. Grossman A. (2018). Transsphenoidal surgery for pituitary tumours: frequency and predictors of delayed hyponatraemia and their relationship to early readmission. Eur. J. Endocrinol. 178, 247–253. doi: 10.1530/EJE-17-0879, 29263154

[ref12] Lee C.-C. Wang Y.-C. Liu Y.-T. Huang Y. C. Hsu P. W. Wei K. C. . (2021). Incidence and factors associated with postoperative delayed hyponatremia after transsphenoidal pituitary surgery: a meta-analysis and systematic review. Int. J. Endocrinol. 2021, 1–13. doi: 10.1155/2021/6659152, 33936198 PMC8055398

[ref13] Lin K. Li J. Lu L. Zhang S. Mu S. Pei Z. . (2021). Diaphragma sellae sinking can predict the onset of hyponatremia after transsphenoidal surgery for pituitary adenomas. J. Endocrinol. Investig. 44, 2511–2520. doi: 10.1007/s40618-021-01611-7, 34128213

[ref14] Lin K. Zeng R. Mu S. Lin Y. Wang S. (2022). Novel nomograms to predict delayed hyponatremia after transsphenoidal surgery for pituitary adenoma. Front. Endocrinol. 13:900121. doi: 10.3389/fendo.2022.900121, 35837309 PMC9273860

[ref15] Lin K. Zeng R. Pei Z. Mu S. Yang Y. Fan Y. . (2021). The difference between preoperative and postoperative pituitary stalk deviation angles can predict delayed hyponatremia after Transsphenoidal surgery. World Neurosurg. 155, e637–e645. doi: 10.1016/j.wneu.2021.08.117, 34481103

[ref16] Miller N. E. Rushlow D. Stacey S. K. (2023). Diagnosis and management of sodium disorders: hyponatremia and hypernatremia. Am. Fam. Physician 108, 476–486. Available at: https://www.aafp.org/pubs/afp/issues/2023/1100/sodium-disorders-hyponatremia-hypernatremia.html 37983699

[ref17] Roncaroli F. Giannini C. (2025). Posterior pituitary tumors and other rare entities involving the pituitary gland. Brain Pathol. 35:e13307. doi: 10.1111/bpa.13307, 39350562 PMC11669417

[ref18] Rutland J. W. Pawha P. Belani P. Delman B. N. Gill C. M. Brown T. . (2020). Tumor T2 signal intensity and stalk angulation correlates with endocrine status in pituitary adenoma patients: a quantitative 7 tesla MRI study. Neuroradiology 62, 473–482. doi: 10.1007/s00234-019-02352-4, 31925468 PMC7205143

[ref19] Skinner D. C. (2009). Rethinking the stalk effect: a new hypothesis explaining suprasellar tumor-induced hyperprolactinemia. Med. Hypotheses 72, 309–310. doi: 10.1016/j.mehy.2008.08.030, 19028420 PMC2668659

[ref20] Snyder M. H. Asuzu D. T. Shaver D. E. Vance M. L. Jane J. A. (2022). Routine postoperative fluid restriction to prevent syndrome of inappropriate antidiuretic hormone secretion after transsphenoidal resection of pituitary adenoma. J. Neurosurg. 136, 405–412. doi: 10.3171/2021.1.JNS203579, 34330096

[ref21] van Furth W. R. de Vries F. Lobatto D. J. Kleijwegt M. C. Schutte P. J. Pereira A. M. . (2020). Endoscopic surgery for pituitary tumors. Endocrinol. Metab. Clin. North Am. 49, 487–503. doi: 10.1016/j.ecl.2020.05.011, 32741484

[ref22] Villa C. Birtolo M. F. Perez-Rivas L. G. Righi A. Assie G. Baussart B. . (2025). Grading and staging for pituitary neuroendocrine tumors. Brain Pathol. 35:e13299. doi: 10.1111/bpa.13299, 39182993 PMC11669418

[ref23] Wu J. Zhang H. Li L. Hu M. Chen L. Xu B. . (2020). A nomogram for predicting overall survival in patients with low-grade endometrial stromal sarcoma: a population-based analysis. Cancer Commun. 40, 301–312. doi: 10.1002/cac2.12067, 32558385 PMC7365459

[ref24] Yoon H.-K. Lee H.-C. Kim Y. H. Lim Y. J. Park H. P. (2019). Predictive factors for delayed hyponatremia after endoscopic transsphenoidal surgery in patients with nonfunctioning pituitary tumors: a retrospective observational study. World Neurosurg. 122, e1457–e1464. doi: 10.1016/j.wneu.2018.11.085, 30465961

[ref25] Yu S. Taghvaei M. Reyes M. Piper K. Collopy S. Gaughan J. P. . (2022). Delayed symptomatic hyponatremia in transsphenoidal surgery: systematic review and meta-analysis of its incidence and prevention with water restriction. Clin. Neurol. Neurosurg. 214:107166. doi: 10.1016/j.clineuro.2022.107166, 35158166

